# Does Gut Microbial Methylglyoxal Metabolism Impact Human Physiology?

**DOI:** 10.3390/antiox14070763

**Published:** 2025-06-21

**Authors:** Oluwatomisono I. Akinrimisi, Kim Maasen, Jean L. J. M. Scheijen, Ina Nemet, Max Nieuwdorp, Casper G. Schalkwijk, Nordin M. J. Hanssen

**Affiliations:** 1 Department of Experimental Vascular Medicine, Amsterdam Cardiovascular Science Institute, Amsterdam University Medical Center, location AMC, 1105 AZ Amsterdam, The Netherlands; o.i.akinrimisi@amsterdamumc.nl (O.I.A.); m.nieuwdorp@amsterdamumc.nl (M.N.); 2 CARIM School for Cardiovascular Disease, Maastricht University, 6229 ER Maastricht, The Netherlands; kim.maasen@wur.nl (K.M.); j.scheijen@maastrichtuniversity.nl (J.L.J.M.S.); c.schalkwijk@maastrichtuniversity.nl (C.G.S.); 3 Department of Internal Medicine, Maastricht University Medical Center, 6229 ER Maastricht, The Netherlands; 4 Department of Cardiovascular and Metabolic Sciences, Cleveland Clinic Lerner Research Institute, Cleveland, OH 44195, USA; nemeti@ccf.org; 5 Center for Microbiome & Human Health, Cleveland Clinic, Cleveland, OH 44195, USA; 6 Diabeter Center Amsterdam, 1066 EC Amsterdam, The Netherlands

**Keywords:** methylglyoxal, microbiome, cardiometabolic disease, ageing, diabetes

## Abstract

Methylglyoxal (MGO) is a highly reactive dicarbonyl associated with oxidative stress, inflammation, and chronic diseases, particularly diabetic vascular complications and atherosclerosis through the formation of advanced glycation end products (AGEs). In the setting of human/host diseases, the formation of MGO has mainly been considered as the byproduct of glycolysis. Gut microbes play an important role in the development of cardiometabolic diseases. Here, we discuss a possibility that gut microbes can modulate the MGO pool within the host through (i) the alternation of the host metabolism, and (ii) direct MGO synthesis and/or detoxification by human commensal microorganisms. We also explore how dietary MGO impacts the composition of the gut microbiota and their potential role in modulating host health. This paradigm is highly innovative, with the current literature providing observations supporting this concept. Targeting the gut microbiome is emerging as an approach for treating cardiometabolic diseases through dietary, pre-, pro-, and postbiotic interventions, faecal microbiota transplantations, and the use of small molecule inhibitors of microbial enzymes. This can be a novel strategy to reduce MGO stress in the setting of cardiometabolic diseases and lowering the burden of diabetic complications and cardiovascular disease.

## 1. Introduction

Methylglyoxal (MGO) is a highly reactive dicarbonyl that reacts with nucleophilic protein and DNA residues and forms advanced glycation end products (AGEs) that contribute to cell and tissue dysfunctions [1]. Within the host, MGO is formed by the spontaneous degradation of glycolytic intermediates. MGO is further metabolised by the evolutionarily conserved glyoxalase system [2,3], which converts MGO to D-lactate. The formation of MGO in human cells and its physiological implications have been well-documented in previous reviews [1,4]. Gut microbes have gained recognition for their important role in maintaining human health. Large cohort studies have repeatedly reported perturbations of the gut microbial community in a context of cardiometabolic diseases [5,6]. Microbial transplantation from mice with glucose intolerance into germ-free mice induce glucose intolerance [7]. In a human faecal microbiota transplant study, a faecal transfer from lean donors into individuals with metabolic syndrome resulted in an increase in gut microbial diversity and improved insulin sensitivity [8]. These data support the hypothesis that the dysbiosis of the gut microbiome contributes to metabolic dysfunction; however, how gut microbial alterations contribute to the metabolic diseases is still not fully understood. Here, we discuss the potential role of gut microbes in influencing the host’s pool of MGO through several mechanisms. Gut microbes may modulate the host MGO/AGE pool by altering the host metabolism. Additionally, microbes, including several human commensals, possess enzymatic capacities to either synthesise or degrade MGO [9,10,11,12,13,14], thereby directly affecting its production and/or detoxification. We also explore how dietary MGO impacts the overall composition of the gut microbiota and their potential role in modulating host health.

## 2. Host Production of Methylglyoxal: Linking Transient Increases in Glycolysis to Long-Term Cellular Dysfunction

In human cells, MGO is predominantly produced by the spontaneous degradation of the glycolytic intermediates glyceraldehyde-3-phosphate (G3P) and dihydroxyacetone phosphate (DHAP). The MGO content in food and beverages is relatively low, typically accounting for less than 1% of total human MGO exposure [1,15], and ~3% comes from the catabolism of threonine [1,16] and ~10% from the degradation of glycated proteins [1,17], but the majority of human MGO exposure, ~90%, is from endogenous formation during glycolysis, with most (~98%) being detoxified by the glyoxylase system in the cells under normal conditions [1,18]. Approximately 0.1–0.4% of the total glycolytic flux results in MGO formation [1,19,20,21,22] and is reported to be formed intracellularly at concentrations of 1–4 μM [16,23,24]. MGO is converted into D-lactate by the glyoxalase system composed of two enzymes, glyoxalase 1 (GLO1) and glyoxalase 2 (GLO2). GLO1, a rate-limiting enzyme, converts hemithioacetal spontaneously formed from MGO and reduced glutathione into S-D-lactoylglutathione. S-D-Lactoylglutathione is then hydrolysed by GLO2 to D-lactate with the accompanied regeneration of glutathione [25]. MGO that escapes the glyoxalase system spontaneously, non-enzymatically, reacts with proteins and DNA, changing their structure and function through the formation of AGEs. MGO reacts primarily with arginine guanidino residues and forms Nδ-(5-hydro-5-methyl-4-imidazolon-2-yl) ornithine (MG-H1), argpyrimidine, and tetrahydroimidazolone (THP), as well as with lysine amino group to form Nε-(carboxyethyl)lysine (CEL). N2-(1-Carboxyethyl)-2′-deoxyguanosine (CEdG) is a major MGO-derived DNA adduct [26]. The increased formation of MGO, thus, leads to the erosion of the intracellular milieu by modifying proteins and DNA, impairing normal cellular functions. Furthermore, MGO is thought to exert epigenetic effects by the direct modification of histones at critical lysine and arginine residues involved in both the nucleosome stability and reader domain binding altering gene expression [27]. Additionally, the induction of MGO production through hyperglycemia induced epigenetic changes in the promoter of the NF-κB p65 subunit in both cultured human aortic endothelial cells and in nondiabetic mice [28]. The accumulation of MGO can further impair cell energy homeostasis via mitochondrial dysfunction [29], the induction of oxidative stress, and the impaired availability of nitric oxide [30]. The clearance of these cellular modifications and impairments is further hampered by the modification of key proteasomal subunits by MGO [31]. Together, these processes may lead to a cycle of the accumulation of MGO and the build-up of modified proteins. The overexpression of *Glo1* in bovine endothelial cells reduced intracellular hyperglycemia and the formation of AGEs [32], indicating that MGO is a major precursor in the formation of AGEs. Higher levels of MGO also drive *GLO1* expression via its upstream regulator nuclear factor erythroid 2-related factor 2 (Nrf2) [33]. The mechanism linking glycolysis to glyoxalase activity has been partly unravelled, wherein MGO modifies KEAP1 to form a complex that enhances the transcription of NRF2 [34].

### 2.1. Infections—An Emerging Inducer of Methylglyoxal Stress

TNF alpha (Tumor Necrosis Factor alpha) is a pro-inflammatory cytokine, involved in the immune response and produced by macrophages. TNF alpha reduced the glyoxalase activity in cultured macrophages [35] and fibrosarcoma cell line L929 through the induction of GLO1 phosphorylation by protein kinase A (PKA), reducing its activity and the concomitant accumulation of MGO to cytotoxic levels [36]. Given its cytotoxic properties, the accumulation of MGO may aid in the inflammatory functions of TNF alpha, and we speculate that the production of MGO by the host serves as an antimicrobial agent during bacterial infections [37]. This is supported by a study demonstrating an increased MGO production during a *mycobacterium* infection of macrophages [38]. Elevated MGO levels have also been observed in individuals with septic shock [39,40], a condition characterised by a dysregulated immune response to infection. Furthermore, the glyoxalase system has been proposed as a defence mechanism in *Group A Streptococcus* against MGO produced by neutrophils [41]. Additionally, MGO detoxification has been shown to function as a virulence mechanism in *Salmonella typhimurium* [42] and *Listeria monocytogenes* [43], indicating that pathogens possess systems to counteract host-produced MGO [43].

### 2.2. How Essential Is Glyoxalase 1 for Maintaining Methylglyoxal Levels?

Drosophila *Glo1* knockouts exhibited elevated MGO levels, increased insulin resistance, and greater weight gain, and developed hyperglycemia, although they remained viable [44]. In zebrafish, *Glo1* deficiency did not alter normal development, likely due to the upregulation of compensatory pathways such as aldehyde dehydrogenase, and showed an enhanced sensitivity to insulin resistance and vascular injury in the retina [45]. *Glo1* knockouts in Schwann cells showed no increase in MGO; there was an alternative detoxification of MGO by aldose reductase [46]. In addition, *Glo1* knockout mice were susceptible to acetaminophen-induced hepatotoxicity [47]; *Glo1* knockdown mice had a diabetic-nephropathy-like phenotype [48], while the manipulation of GLO1 activity did not alter the atherosclerotic burden in atherosclerosis-prone Apoe-/- mice [49]. These findings suggest that MGO plays a role in modulating the organismal physiology and reveal the complexity of its detoxification.

## 3. Methylglyoxal and the Microbiome

**Figure 1:**
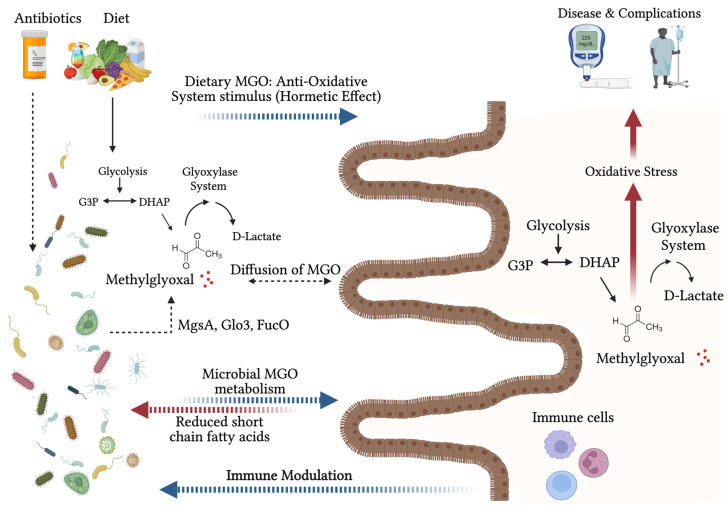
Illustration depicting the fate of MGO in human gut microbiome interactions. Red arrows indicate detrimental consequences, blue arrows indicate beneficial effects, solid black arrows represent pathways, and dashed arrows illustrate suggestive factors that may influence MGO buildup or loss. MgsA (methylglyoxal synthase), Glo3 (glyoxylase 3), FucO (1,2 propanediol oxidoreductase), G3P (glyceraldehyde 3 phosphate), DHAP (dihydroxyacetone phosphate).

The interplay between MGO, the microbiome, and the host is complex. Although it has not been investigated definitively, various works in the literature elude to the concept that the microbiome may have a role to play in influencing the gut MGO pool and, in turn, systemic MGO levels, either through the diffusion of MGO across the intestinal barrier, or in a case where a disturbed intestinal barrier integrity allows microbial toxic compounds to enter the blood stream or, in fact, the microbes themselves [50,51]. Firstly, certain microbes possess specialised MGO metabolism genes with functions not found or demonstrated in humans such as methylglyoxal synthase (*mgsA*), glyoxylase 3 (*glo3*), and 1,2-propanediol oxidoreductase. Second, as described in Section 2.1, MGO may serve as a mediator between microbes and immune cells, particularly during infections. Third, SCFAs, which are microbial metabolites, have been shown to modulate MGO levels, and MGO itself may influence SCFA production [14,52,53]. Additionally, the dietary MGO present in various food and beverages interacts with gut microbes. Given the microbe’s role in modulating the host digestion and metabolism, the microbiome may indirectly regulate MGO concentrations in the host. Subsequent sections will explore the relevant literature regarding microbial communities and host MGO metabolism in more detail. Figure 1 illustrates the potential fates of MGO within the context of the host gut microbiome interactions.

**Figure 2:**
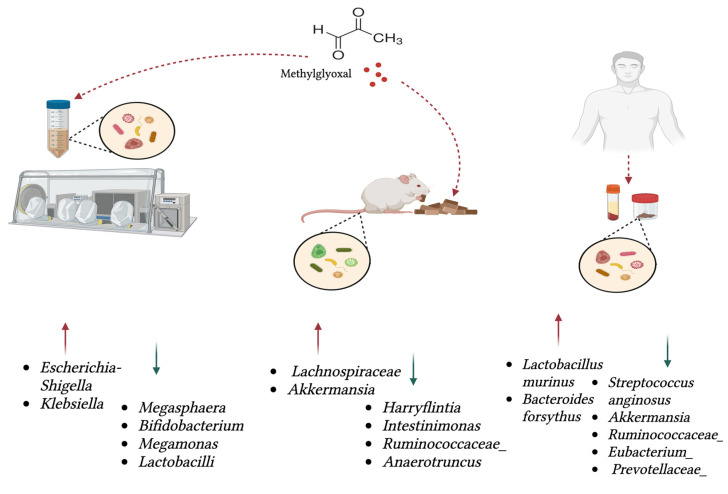
Illustration summarizing (Table 1), showing microbes that increase or decrease in abundance in response to MGO. Red arrows are positive associations with MGO while green arrows are negative associations.

Unlike in the host where MGO is produced spontaneously, i.e., non-enzymatically, microbes, including multiple commensals, produce MGO through the catalytic dephosphorization of dihydroxyacetone phosphate, by MgsA encoded by the *mgsA* gene [54]. Another microbial pathway in the formation of MGO is from aminoacetone generated from glycine and threonine via the oxidoreductase [55,56,57]. The microbial MGO metabolism has been demonstrated primarily through experiments using microbes grown in culture media [9,58,59,60,61,62,63,64]. Relatively few studies have explored the broader impact of MGO on the microbiome as a whole; these are summarised in Table 1 and Figure 2. There is limited overlap between studies, with studies also having differing methodologies and research objectives (Table 1). Microbial taxa show a positive association with MGO, with increased abundance, while others display a negative association (Table 1). We hypothesise that a positive association is due to intrinsic tolerance or the ability to metabolise MGO, whereas negative associations may arise from a lack of tolerance to MGO or from competitive exclusion by other microbes that are better adapted to utilise MGO as a resource within the microbial community. Across both clinical and experimental studies, MGO was shown to selectively influence the composition of the microbiome. Several taxa such as *Ruminococcaceae* [65,66], *Bifidobacterium* [52], and *Lactobacilli* [53] showed negative associations with MGO exposure, while potentially pro-inflammatory or opportunistic genera like *Escherichia-Shigella*, *Klebsiella* [52], and *Bacteroides forsythus* [67,68] tend to be enriched. *Akkermansia* displays context-dependent behaviour, appearing as both positively [65] and negatively [66] associated with MGO depending on the host condition and study model. While the in vivo data from aged mice revealed no significant changes in the overall alpha or beta diversity [65], the in vitro studies report both marked compositional shifts in microbial abundance [53] and demonstrated microbial differences using principal coordinate analysis [52]. These findings suggest that MGO does not necessarily alter global diversity metrics but can drive substantial taxonomic and functional shifts. We also believe that such changes are likely dependent on the dose of MGO used. Clinically, elevated MGO levels have been associated with conditions like rectal cancer [66] and periodontitis [67,68], where they correlate with specific microbial signatures, indicating that MGO may act not only as a microbial modulator but also as a potential biomarker, with the disease-related microbial shifts requiring further investigation.

Short-chain fatty acids (SCFAs), including the popular acetate, propionate, and butyrate, are important microbial metabolites. In *Proteus* and *Morganella* spp., the addition of butyric and propionic acids has been shown to reduce MgsA activity and decrease the MGO concentration in the culture medium [14]. SCFAs are produced by the gut microbe fermentation of dietary fibers; conditions present in the gut shaped by the already colonizing bacteria could influence the microbial production of methylglyoxal [14]. In the other direction, evidence also indicates that MGO may impact SCFA production. In in vitro fermentation using human faecal samples, the supplementation of MGO led to a reduction in SCFA levels for acetate, propionate, and butyrate [52]. A study simulating the digestion of dicarbonyl compounds in vitro and using faecal samples from three human donors demonstrated that MGO exposure resulted in diminished SCFA production [53]. Taken together, these findings suggest that MGO in high concentrations may exert detrimental effects on the gut microbiome, by reducing the number of beneficial bacteria capable of SCFA production.

### 3.1. Theories on Why Microorganisms Produce Methylglyoxal

Given the fact that MGO is cytotoxic, its active production by microbes seems counterintuitive. One hypothesis to explain this is that MGO production is a microbial adaptation to nutrient imbalance, particularly under conditions of excess carbon availability. In such environments, key glycolytic intermediates may accumulate to toxic levels, disrupting normal metabolic function [69,70]. MGO may act as a metabolic overflow pathway, helping to regulate the carbon flux and prevent the buildup of glycolytic intermediates [54,69]. Although MGO may be inherently toxic to the cell, microbes possess active protective mechanisms such as the glyoxalase system that converts MGO into less harmful compounds like D-lactate. This detoxification strategy allows microbes to temporarily tolerate MGO while benefiting from its role in maintaining the metabolic balance [69]. Overall, the hypothesis suggests that MGO production offers a short-term advantage during environmental shifts, when microbes encounter nutrient-rich but imbalanced conditions. Another theory is focused on the regulation of phosphate availability. MgsA converts DHAP into MGO, releasing phosphate in the process. This phosphate can then be utilised in the glyceraldehyde-3-phosphate dehydrogenase reaction during glycolysis [54]. Thus, MgsA may play a role in generating phosphate for glycolytic processes [54]. Moreover, the Catabolite Repression HPr-like protein (Crh) was found to regulate MgsA activity in *Bacillus subtilis*, with its non-phosphorylated form binding to and inhibiting MgsA [71]. Preferred carbon sources such as glucose or mannitol trigger the phosphorylation of Crh and reduce its binding to MgsA [71], suggesting that MGO is produced when the preferred carbon sources are present [71]. Lastly, the mechanisms of microbial cross-feeding in the gut are well-highlighted [72]; cross feeding involves the sharing of metabolites, including 1,2-propanediol [72] between microbes. In such systems, the metabolites of one microbe are released and utilised by another, fostering interactions that range from mutualism to competition. From a microbial ecological perspective, we speculate some microbes may release MGO into the environment to other microbes or as a way to inhibit the growth of other microbes. A metabolic flux analysis in *Escherichia coli* reveals a dynamic response to changing nutrient conditions, “famine” (increased glucose availability) and “feast” (decreased glucose availability). During feast conditions, the excretion of MGO and lactate occurred rapidly, followed by acetate at a later stage [73]. MGO levels in the medium reached 0.6–0.9 mg/L [73], well below the reported threshold of 20 mg/L that can inhibit microbial growth [73]. In contrast, under famine conditions, no byproducts were detected in the culture medium. This indicates that the MGO pathway is tightly regulated in *Escherichia coli*; even the overexpression of *mgsA* caused the accumulation of only ~0.08 mM MGO in the glucose medium [74], *Escherichia coli* is reported to lose viability at 0.6–0.7 mM MGO [69]. In *Saccharomyces cerevisiae,* MGO accounts for 0.3% of the glycolytic flux [62].

### 3.2. Microbial Detoxification of Methylglyoxal

Similar to the host MGO detoxification routes, bacteria also have a glyoxalase system responsible for the detoxification of MGO into D-lactate. Furthermore, another route identified in *Escherichia coli* is the glutathione-independent glyoxalase III (Glo3) where MGO is converted directly to D-lactate and is glutathione-independent [75]. Glo3 has primarily been characterised and its activity demonstrated in *Escherichia coli* [76,77]. The *hchA* gene, which encodes Hsp31 (Glo3), is important for MGO resistance and Hsp31 is a DJ-1 homolog that is widely evolutionarily distributed [76]; *hchA*-deficient strains of *Escherichia coli* in the stationary phase showed an increased sensitivity to MGO and accumulated higher levels of intracellular MGO [77]. The first physiologically relevant demonstration of glutathione-independent Glo3 activity in fungi was reported in *Candida albicans*, where the homozygous *glx3* knockout mutants displayed sensitivity to exogenous MGO, and had increased intracellular MGO, and carbon-source-specific growth defects [78]. In contrast, GLO3 has not yet been identified in humans [79]. Other conversion routes include the enzymes alcohol dehydrogenase and methylglyoxal reductase [69,80], although the physiological relevance of these enzymes in *Escherichia coli* is questionable due to their low affinities for MGO [69]. Additionally, we have glycerol dehydrogenase, which converts MGO to D-lactaldehyde, and 1,2-propanediol oxidoreductase, which converts L-lactaldehyde to 1,2-propanediol [76]. A variety of microbes have been reported to produce 1,2-propanediol [81,82,83]; however, the conditions and substrate for production vary, with varied pathways for 1,2-propanediol production including the deoxyhexose pathway, lactate pathway, and MGO pathway described in this review [82]. The MGO pathway has been described previously where sugars such as glucose and fructose are substrates for glycolysis and MgsA converts the glycolytic intermediate DHAP into MGO. MGO is subsequently reduced to 1,2-propanediol through D-lactaldehyde in the presence of glycerol dehydrogenase, and then 1,2-propanediol oxidoreductase, or through acetol in the presence of alcohol dehydrogenase, and then glycerol dehydrogenase [82]. In *Clostridium thermosaccharolyticum*, phosphate concentrations of up to 113 mM had no significant effect on product (1,2-propanediol/acetol) formation through the MGO pathway [84], contrary to other reports where MgsA is inhibited by the presence of phosphate [54,85]. Additionally, *Escherichia coli* activates the potassium efflux systems KefB and KefC in response to the formation of a glutathione conjugate [69]. Homologs of KefC have been identified in other Gram-negative bacteria, including *Salmonella typhimurium* and *Enterobacter aerogenes*. This system appears to be absent in Gram-positive bacteria [69,86], with the exception of *Staphylococcus aureus* [86]. These systems may help protect the cell from MGO toxicity by exporting potassium ions out of the bacterial cell [87,88], accompanied by an influx of sodium ions and protons. This ion exchange leads to cytoplasmic acidification, resulting in a lower intracellular pH [89,90]. The proposed model suggests that this acidification reduces the harmful interactions between MGO and macromolecules, and may also stimulate DNA and protein repair mechanisms [69].

### 3.3. The Microbiome as a Potential Therapeutic Target to Lower Methylglyoxal Stress in the Host

To date, no known mice or human studies have investigated altering the gut microbiome using probiotic strains, commensal microbes, or faecal microbiota transplantation (FMT) with the specific aim of reducing systemic MGO levels in the host, although the influence of the microbiome on host health and metabolism is of increasing interest [91,92]. The microbiome has been reported to influence the host energy intake [93], metabolism of macronutrients [91] and micronutrients [94], dictate the appetite via the brain–gut axis [95,96], and modulate the immune system [97]. Microbes can also influence host metabolite levels which have diverse effects on the host [98,99]. SCFAs, produced by gut microbes from dietary fiber, are recognised as beneficial metabolites that help restore intestinal barrier integrity [100], support blood glucose regulation, enhance insulin sensitivity in individuals with type 2 diabetes [101,102], and carry out the regulation of energy homeostasis, and body weight control [103,104]. Thus, we hypothesise that the microbiome may influence MGO stress via host metabolic regulation, whereby a state of microbial dysbiosis negatively impacts the host metabolism. As previously mentioned, microbes possess several enzymes for MGO detoxification (Glo1, Glo2, and Glo3) and MGO synthesis (MgsA). Thus, the gut microbiome may act as a reservoir for MGO, allowing its entry into systemic circulation via the portal vein. Targeting the microbiome through supplementation with beneficial strains may help mitigate MGO-related stress. *Lactobacillus* spp. have gained popularity as probiotics due to their associated health benefits [105]. Interestingly, *Lactobacilli* sp. have been shown to convert MGO into 1,2-propanediol [9], while, in a mouse study, *Lactobacilli* were found to potentially prevent liver injury by stimulating the antioxidant pathway Nuclear factor Nrf2 [106], which is reported to help reduce MGO stress by promoting a GSH increase [107], and regulate the expression of *GLO1* [33]. This suggests that the microbiome may function as a first line of defense against exogenous MGO, either by converting it into other metabolites or by directly neutralizing it. Conversely, in cases of gut dysbiosis, there may be an increase in MGO-producing proteins or enhanced MGO production by immune cells, which could result from an imbalance in microbial populations. For instance, the gut microbial production of MGO by *Escherichia coli* accelerated ageing in *Caenorhabditis elegans* [108], demonstrating that microbial MGO can directly impact the host physiology. Further research is needed in order to explore these hypotheses and advance our understanding for the greater good.

## 4. Dietary Methylglyoxal

Besides endogenous formation, we are also exposed to MGO through exogenous sources, mainly through diet. MGO is formed during food processing and preparation, as part of non-enzymatic browning (Maillard reaction), the caramelization reaction, and lipid peroxidation, or enzymatically by microorganisms in fermented foods [109]. The presence of MGO in foods and drinks has been evaluated in several studies, the most extensive of which is a comprehensive food composition database with an MGO content of 223 commonly consumed products quantified using the gold-standard UHPLC-MS/MS technique, covering a broad range of food groups, including vegetables, fruit, cheese, meat, fish, and ready-made products [110]. In this database, MGO concentrations ranged from 0.04 to 736 mg/kg, and were the highest in manuka honey, dried apricot, Dutch spiced cake, rusk, apple molasses, and digestive biscuit. MGO concentrations are generally high in fermented foods [15] and foods containing caramel or molasses (both rich in sugar and exposed to intense heat treatment). Several factors that impact dicarbonyl formation in foods have been described, i.e., the type of sugar used, the intensity of heat treatment and the accompanying reduction in water content, food processes such as ripening, fermentation, and storage, and the absence of reactants like amino acid side chains of proteins that may result in the subsequent formation of AGEs and, thus, reduce the dicarbonyl content [15]. In the Dutch population, MGO intake comes mainly from coffee consumption, followed by bread, and cookies/bakery products [110]. As stated previously, the MGO content in food and beverages is relatively low, typically accounting for less than 1% of total MGO exposure [1,15]. Several studies have attempted to perform the simulated digestion of MGO, yielding differing results [111,112,113]; simulated digestion experiments are different from actual conditions in the gastrointestinal tract [112]. Upon simulated in vitro gastrointestinal digestion, 5–20% of the initial MGO was recovered [114]; in addition, four healthy volunteers on a controlled diet were administered 500 μmol of MGO in manuka honey, and the renal excretion of MGO ranged from 0.1 to 0.4 μmol/day [114]. This study suggests that dietary MGO undergoes rapid degradation during intestinal digestion and, consequently, does not affect in vivo MGO levels [114]. On the contrary, in vitro gastrointestinal digestion (oral, gastric, and small intestinal phases) in another study reported ~75% intact MGO where they make it to the colon and can react with microbes [53], while a cross-sectional study in the Netherlands (The Maastricht Study) investigated the relationship between the habitual intake of MGO using dietary data. The study found that a higher habitual MGO intake was associated with lower levels of low-grade inflammation [115].

### 4.1. Methylglyoxal in Manuka Honey

Manuka honey is a honey derived from *Leptospermum scoparium* trees, which is known for its various therapeutic beneficial properties and very high MGO content [116,117], compared to other types of honey [118]. MGO has been reported to be responsible for the antimicrobial activity of manuka honey [118,119]. MGO derived from manuka honey has been shown to inhibit bacterial growth and prolong the lag phase of various bacterial species [120]. Manuka honey has also been shown to inhibit biofilm formation in bacterial species [121,122]. It has been reported that MGO from manuka honey may contribute to host mucosal-associated invariant T-cell-sensing microbes [123], which would be beneficial for immune homeostasis. MGO exhibits antibacterial activity against Gram-positive and Gram-negative bacteria [124,125,126]. Additionally, MGO has been shown to be effective against microbial biofilms [119] and multidrug-resistant strains [126], and enhance the susceptibility of microbes to antimicrobial agents [127,128]. MGO may prevent microbial growth through the damage of the structural integrity of bacterial flagella and fimbriae, leading to a loss of their function [124]. However, a mouse study reported that the dietary intake of manuka honey, which is rich in MGO, did not cause substantial alterations in the composition of the gut microbiota or the levels of microbial-derived SCFAs [129]. Although the authors of the study acknowledge some methodological limitations, for instance, the next-generation sequencing-based phylogenetic analysis may have identified more microbial taxa than the RT-qPCR method used in the study; conducting further research through stable isotope tracing and a flux analysis of the organic acid uptake, in addition, would have been beneficial. Lastly, in their study, manuka honey delivers ∼0.12 mM MGO [129]; they suspect higher concentrations may be required to show antimicrobial effects due to the loss of MGO during digestion processes [129].

### 4.2. Metabolism of Methylglyoxal in the Gut

MGO is a small compound, and it is conceivable that it is absorbed from foods in the intestine. Animal studies reported increased plasma concentrations of MGO after oral MGO administration via drinking water [130,131,132]. In humans, a small-scale study reported that a single oral dose of manuka honey containing a high amount of MGO (i.e., ~500 μmol) was given [114]. This single dose of MGO did not lead to changes in urinary MGO excretion, but no data on plasma MGO or AGEs was available. A recent human population cohort examined the habitual intake of MGO, and observed that a higher habitual intake of dietary MGO was associated with higher plasma concentrations of MGO and a higher skin autofluorescence, an estimate of skin AGEs that is thought to reflect AGEs in tissue [133]. Coffee and bread were the main dietary sources of MGO in this cohort, and the associations of dietary MGO with plasma MGO and with skin autofluorescence were mainly driven by coffee intake. These results suggest the dietary absorption of MGO, but we cannot exclude the possibility that MGO from the diet indirectly influences MGO in the body. A 1 mg/day higher intake of MGO (corresponding to, for example, ~400 mL coffee [110]) was associated with a 2–3% higher plasma MGO and skin autofluorescence. Notably, the concentrations of free MGO in (fasting) plasma might provide only a snapshot and be an underestimation of the actual contributions of dietary MGO. Besides the dietary absorption of MGO, some of the MGO from foods may react with proteins present in the gastrointestinal tract, for example, with proteins present in the food matrix [113,114,133], with digestive enzymes [134], or with intestinal proteins [111]. The modification of food proteins could lead to the de novo formation of AGEs, which can, thereafter, be absorbed or exert local effects in the gastrointestinal tract. The modification of digestive proteins may lead to altered digestion, whereas the modification of intestinal proteins may lead to gut inflammation, increased permeability, or an altered microbiome composition [109,135,136,137]. The reactivity of MGO during digestion has, so far, only been studied in vitro. Studies with human intestinal cells reported that MGO induced an increased secretion of the pro-inflammatory cytokines interleukin-6 (IL-6) and interleukin-8 (IL-8) [138,139]. A decrease in MGO concentration was observed in several studies during simulated gastrointestinal digestion [114,140,141]. However, an increase in MGO concentration has also been reported in soy sauce, possibly due to their release from reversibly-bound adducts [140]. This indicates that reactions during digestion may play an important role in the actual exposure to MGO throughout the gastrointestinal tract. It is important to note here that a reduction in MGO does not mean that they are harmless, per se, since the putative consequences of the reaction-products are unknown. Furthermore, it should be emphasised that these models might not represent in vivo digestion. Moreover, when investigating the gastrointestinal metabolism of MGO, it is essential that we consider the food matrix in which MGO is present. The release may be modulated by the complexity of the food matrix, or the presence of scavengers of MGO in the food matrix. Examples of potential scavengers are creatinine in meat [142], fiber-bound polyphenols from highland barley [52], and melanoidins in coffee, cacao, and bread, for which MGO-scavenging effects have been reported in vitro [143]. Thus, it is important that we consider the food matrix when predicting the effects of dietary MGO.

### 4.3. Consequences of Dietary Methylglyoxal

Studies in animals showed that high amounts of orally administered MGO increased vascular inflammation [131,144] and induced vascular changes such as impaired vasodilation [131,145], the thickening of the glomerular basement membrane [146], endothelial dysfunction [131], the induction of retinopathy-like changes, [147], and insulin resistance [148]. Moreover, the administration of exogenous MGO, either subcutaneously or intraperitoneally, induced β-cell dysfunction, insulin resistance and impaired glucose metabolism, and insulin sensitivity [149,150]. In contrast, a theory suggesting that MGO has beneficial effects within a hormetic model has been proposed, where MGO has harmful effects at high doses and beneficial effects at low doses [25]. A recent study in mice showed that the long-term exposure to orally administered MGO resulted in a moderate increase in plasma MGO; yet, this was accompanied by increased survival, less age-related solid tumors, and the absence of diabetes and renal insufficiency [130]. Moreover, in rats, a similar increase in plasma MGO after long-term administration with oral MGO was observed, but no differences were reported in oxidative stress and higher levels of antioxidants [132]. Similarly, the anti-oxidative effects of low doses of MGO were reported in vitro [34,151,152]. A possible explanation for this is that small increases in plasma MGO via exogenous intake may have beneficial effects, for example, through the upregulation of the antioxidant system and/or the glyoxalase system, a major pathway in the detoxification of MGO [151]. A large, cross-sectional cohort study in The Netherlands (The Maastricht Study) explored associations between habitual MGO intake (by matching the dietary data from the FFQ with our food composition database, which include the concentrations of MGO, GO, and 3-DG in more than 200 foods) and several health outcomes. Unexpectedly, a higher habitual intake of MGO was associated with lower low-grade inflammation scores [115]. Moreover, in the same cohort, a higher habitual MGO intake was associated with greater insulin sensitivity and less type 2 diabetes [153]. There were no consistent associations between MGO intakes and β-cell function. Possible explanations for the discrepancies between these studies in animals and humans are that experimental studies were often conducted with very high concentrations of MGO, out of the physiological range, whereas the exposure to MGO in humans is much lower. In addition, most studies in animals used a commercially available MGO, often contaminated with other reactive compounds. Additionally, it is also important to consider the food matrix when evaluating the role of dietary/exogenous MGO on the host. The associations between dietary MGO and health outcomes were mainly driven by coffee intake, thus far the largest identified contributor of MGO intake from the diet [153]. However, the effects of MGO in coffee cannot be disentangled from that of other compounds in coffee. Therefore, intervention studies with proper food matrix controls are needed in order to evaluate the role of dietary MGO on the host. Overall, these findings suggest that not only does MGO have harmful effects, but the consumption of dietary MGO might lead to small increases in plasma MGO, which may induce some beneficial effects.

## 5. Consequences of Methylglyoxal Stress

To date, no known mice or human studies have attempted to determine the proportional contributions of microbially derived MGO to the systemic circulation separate from MGO produced endogenously by the cells of the host. The predominant body of experimental literature has focused on either the exposure to (very) high levels of exogenous MGO or the manipulation of the glyoxalase system and quenchers of MGO in severe models of experimental diabetes, well-described in other reviews [1,4]. Therefore, we summarise the emerging evidence for the role of MGO in diabetic complications as well as other vascular and age-related conditions, and we provide hypotheses for how the manipulation of the microbiome may influence MGO stress.

### 5.1. Methylglyoxal and “Classic” Microvascular Diabetic Complications—Its Mechanisms of Action

MGO has been associated with neuroretinal dysfunction [147]; feeding rats 50 mM MGO in drinking water induced a retinopathy/like phenotype [147]. In line with this, the manipulation of intracellular MGO formation, by overexpressing *Glo1*, reduced diabetic retinopathy and nephropathy in rats [30,154]. A mouse study suggests that TRPC channels play a role in the accumulation of MGO, with the subsequent *TRPC* knockout mice protected against diabetic retinopathy, having lower MGO levels and increased GLO1 activity [155]. The gut–retina axis is a growing field of research, where the microbiota may play a role in disease severity. A study showed that feeding mice a diet of high glycemic load led to the acceleration of macular degeneration with the accumulation of AGEs; they found that bacterial species were associated with the pace of damage and protection [156].

### 5.2. Methylglyoxal as an Inducer of Cerebral Ageing and Cellular Senescence

Since endogenous MGO leads to oxidative stress and the formation of AGEs, the formation of MGO has been proposed to be one of the key pathways contributing to the ageing process. In the brain, the most energy-demanding organ with the poorest capacity to regenerate and divide, neurons largely convert lactate produced by astrocytes into pyruvate and acetyl-CoA to bypass the glycolytic steps that lead to the formation of MGO [21]. Such bypasses, however, do not seem to be afforded to peripheral nerves, as MGO is thought to be a key driver of diabetic neuropathy [157]. Senescence is emerging as a key cellular process involved in ageing. Cell senescence is a process where cell cycle arrests, and an associated senescence associated inflammatory signature (SASP) arises, leading to further dysfunction of the surrounding tissue. To what extent MGO induces cellular senescence in the strictest sense is still largely unknown. The gut microbial production of MGO by *Escherichia coli* accelerated ageing in *Caenorhabditis elegans* [108].

Exposure to MGO induces cell cycle arrest in human umbilical endothelial cells (HUVEC), without a clear increase in inflammatory signaling [158]. In contrast, in a mouse model of accelerated ageing by daily D-galactose injection, cortical MGO accumulation was linked to memory impairment, impaired glyoxalase activity, and increased inflammatory signaling [159]. Unfortunately, a single-cell approach to pinpointing the site of MGO accumulation in the brain is lacking, but older immunohistochemical studies using AGE staining suggest that the astrocytes and microglial cells are the primary cells of MGO accumulation [160], in line with their highest direct glycolytic rate. MGO has also been linked to direct signaling in the brain, either through binding directly to receptors and sodium channels, or through modifying neurotransmitters [161]. These observations together may aid the explanation that diabetes is both linked to depression as well as an increased risk for neurodegenerative disorders. We observed increased MGO-derived AGE levels in the brains of streptozotocin-injected rats, and we postulate that, by inducting endothelial dysfunction, MGO may contribute to the impairment of the blood–brain barrier, leading to further damage to the brain. In line with this hypothesis, *Glo1* overexpression partly rescued the mild cognitive impairment in these rats [162]. Whether these mechanisms could be influenced by circulating MGO derived from excess production in the gut microbiome has not been studied, but it seems overall unlikely. Nonetheless, since the microbiome has established effects on insulin resistance, indirect effects of the gut microbiome on cerebral MGO levels are conceivable. Furthermore, the gut microbiome may produce MGO-quenching or GLO1-inducing compounds that alter MGO levels, but these kinds of potential mechanisms remain largely speculative.

### 5.3. Methylglyoxal and Cardiovascular Disease

Higher MGO levels are linked to an increased risk of cardiovascular events in type 1 diabetes [163] and type 2 diabetes, and in individuals with critical limb ischemia, regardless of diabetes status [164]. In carotid atherosclerotic plaques, we found that it was not the diabetes status but plaque macrophage content that was associated with higher plaque MGO levels [35]. Using immunohistochemical studies with antibodies against MG-H1, we determined the plaque shoulder bordering the necrotic core as the predominant site of MGO accumulation [35]. Oral and gut microbes are associated with plaques of patients with atherosclerosis [165]. Oral commensals have specifically been identified in the atherosclerotic plaques of patients with coronary artery disease [166]. Moreover, microbes can form biofilms in carotid arterial plaques and are believed to contribute to plaque rupture [167]. We previously found that overexpressing *Glo1* failed to reduce the increased MGO levels in streptozotocin-injected mice, suggesting that there may be an additional, extracellular source of MGO, such as diet and/or a perturbed microbiome [168]. We are currently not aware of any antibiotic or faecal microbiota transplantation studies to shed further light on these issues. The complexity of this system is further underlined by a publication showing that either the overexpression or knockdown of *Glo1*-altered albuminuria in Apoe-deficient mice had no effect on atherosclerosis [49]. Therefore, the formation and detoxification of MGO is highly context-dependent. In addition to its effects on arteries, MGO seems to have direct detrimental effects on the cardiac muscle. Harm by MGO has been mostly described in the context of increased intracellular formation in the setting of diabetes, using *Glo1*-overexpressing rodents. One study found that *Glo1* overexpression improved cardiac dysfunction in streptozotocin-injected mice, and that this was mainly due to an improvement in endothelial dysfunction. Largely in line with our atherosclerosis experiment, this study only found a modest reduction in dicarbonyl stress in the hearts of the *Glo1*-overexpressing mice [169].

### 5.4. Methylglyoxal and Cancer

The formation of MGO in cancer has mainly been studied in the setting of increased glycolysis by the so-called Warburg effect [170]. In turn, MGO may accelerate tumor progression by inducing a direct modification of DNA or by the induction of epigenetic signatures. Recently, it was, in fact, shown in breast cancer that MGO can bypass the seminal two-hit tumor suppressor hypothesis by inactivating the *BRCA2* gene product because the MGO-modified protein undergoes proteolysis [171]. The role of exogenous MGO exposure was studied in breast and glioma cells, showing that exogenous MGO (at low doses) induced NRF2 and GLO2, and promoted tumor growth [33]. A small study of 26 individuals with rectal cancer identified MGO as the key metabolite produced by the faecal microbiome, predicting the response to chemotherapy [66]. Compared to wild-type cells, colorectal cancer cells with KRAS mutations survive better under MGO stress through AKT activation. Additionally, MGO stress makes wild-type cells resistant to cetuximab [172]. The link between endogenous and microbial MGO production and cancer is not straightforward. Nonetheless, these cancer studies raise the question as to whether similar interplays also occur in other organs.

## 6. Conclusions

MGO is a ubiquitous compound produced endogenously by the host metabolism and by microbes in the gut, and is present in various dietary sources. A review of the current literature suggests that the effects of MGO are highly context- and dose-dependent, with elevated endogenous exposure more commonly associated with detrimental outcomes. While the mechanisms by which MGO influences cellular physiology have been well-characterised, and the critical role of the microbiome in human health is increasingly recognised, relatively few studies have bridged the gap between MGO biology and host–microbiome interactions. To advance this field, future research should prioritise the use of stable-isotope-labeled MGO to accurately trace its gastrointestinal fate, microbial transformation, and systemic absorption. In addition, in vivo experiments are needed in order to elucidate how microbes interact with MGO within complex microbial communities, rather than in isolated cultures with study designs involving antibiotics, faecal microbiota transplantation, and germ-free mice. Lastly, large-scale population studies examining the associations between dietary MGO intake, circulating MGO levels, and health outcomes will also be critical. While the role of microbially derived MGO in human physiology remains unclear, the tools and methodologies now exist to explore this important frontier in host–microbe interactions.

## Figures and Tables

**Table 1:** Studies investigating the influence of MGO on the microbiome. A visual summary is provided (Figure 2).

Study Summary	Positively Associated with MGO	Negatively Associated with MGO	**Reference**
Oral MGO administration in aged mice caused shifts in specific taxa and metabolic pathways, with no change in alpha or beta diversity; mice were given MGO (100 mg/kg/day) for 4 weeks.	*Lachnospiraceae*, *Akkermansia*	*Harryflintia*, *Intestinimonas*, *Ruminococcaceae Family*, *Anaerotruncus*	[65]
Faecal and serum samples from 26 patients with locally advanced rectal cancer showed that MGO predicts treatment response and correlates with specific microbes.	*Lactobacillus murinus*	*Streptococcus anginosus*, *Akkermansia*, *Ruminococcaceae*, *Eubacterium*, *Prevotellaceae*	[66]
In vitro fermentation of human faecal samples with MGO was analysed using 16S rRNA gene sequencing. Principal coordinate analysis (PCoA) of the microbiome shows separation between the blank group and the MGO group.	*Escherichia-Shigella* and *Klebsiella*	*Megasphaera*, *Bifidobacterium*, *Megamonas*	[52]
Simulated digestion of dicarbonyls and in vitro fermentation with 3 human faecal samples showed that MGO and other 1,2-dicarbonyls reduced gut microbial abundance and activity. Low MGO level used—6.0 mg/kg; high MGO level used—219.6 mg/kg.		*Lactobacilli*	[53]
In 14 patients with chronic periodontitis, MGO accumulated in gingival crevicular fluid at high levels compared to the reference group.	*Bacteroides forsythus*		[67,68]

## Data Availability

Not applicable.

## Abbreviations

AGEs	Advanced glycation end products
DHAP	Dihydroxyacetone phosphate
FFQs	Food Frequency Questionnaires
G3P	Glyceraldehyde-3-phosphate
GLO1	Glyoxalase 1
GLO2	Glyoxalase 2
GSH	Glutathione
MGO	Methylglyoxal
SCFA	Short-chain fatty acids
